# MPET^2^: a multi-network poroelastic and transport theory for predicting absorption of monoclonal antibodies delivered by subcutaneous injection

**DOI:** 10.1080/10717544.2022.2163003

**Published:** 2023-01-10

**Authors:** Hao Wang, Tianyi Hu, Yu Leng, Mario de Lucio, Hector Gomez

**Affiliations:** a School of Mechanical Engineering, Purdue University, West Lafayette, IN, USA; b Weldon School of Biomedical Engineering, Purdue University, West Lafayette, IN, USA

**Keywords:** Subcutaneous injection, multi-network poroelastic (MPET) model, monoclonal antibodies, subcutaneous biomechanics, biomechanical modeling

## Abstract

Subcutaneous injection of monoclonal antibodies (mAbs) has attracted much attention in the pharmaceutical industry. During the injection, the drug is delivered into the tissue producing strong fluid flow and tissue deformation. While data indicate that the drug is initially uptaken by the lymphatic system due to the large size of mAbs, many of the critical absorption processes that occur at the injection site remain poorly understood. Here, we propose the MPET^2^ approach, a multi-network poroelastic and transport model to predict the absorption of mAbs during and after subcutaneous injection. Our model is based on physical principles of tissue biomechanics and fluid dynamics. The subcutaneous tissue is modeled as a mixture of three compartments, i.e., interstitial tissue, blood vessels, and lymphatic vessels, with each compartment modeled as a porous medium. The proposed biomechanical model describes tissue deformation, fluid flow in each compartment, the fluid exchanges between compartments, the absorption of mAbs in blood vessels and lymphatic vessels, as well as the transport of mAbs in each compartment. We used our model to perform a high-fidelity simulation of an injection of mAbs in subcutaneous tissue and evaluated the long-term drug absorption. Our model results show good agreement with experimental data in depot clearance tests.

## 1. Introduction

Therapeutic monoclonal antibodies (mAbs) are widely used in the treatment of a broad range of diseases, including diabetes, rheumatoid arthritis, and cancers (Pisal et al., 2010; de Zwart et al., 2016; Vennepureddy et al., 2017). MAbs have attracted even more attention since the COVID-19 pandemic due to their capability to target the viral spike protein and to prevent binding of the virus to host cells (Chavda et al., 2022; Li & Gandhi, 2022; Lin et al., 2022; Uraki et al., 2022). Because protein-based mAbs experience denaturation in the acid environment of the stomach, they must be administered via parenteral route (Reilly et al., 1997). Among different parenteral administration approaches, subcutaneous injection is a preference for patients and caregivers due to its low cost and low risk of systemic infection (Haller, 2007; Jackisch et al., 2014; Awwad & Angkawinitwong, 2018; Turner & Balu-Iyer, 2018; Viola et al., 2018). In addition, subcutaneous injection is beneficial to patients with poor venous access or in long-term treatment (Launay-Vacher, 2013). Compared to intravenous administration in which the drug is directly injected into the circulatory system, in subcutaneous injection the drug is injected into subcutaneous tissue and absorbed into blood and lymphatic capillaries via inner fluid communications between interstitial tissue and the embedded capillaries. Thus, relatively low bioavalibility is one of the shortcomings of subcutaneous administration (Porter et al., 2001). Although a lot of experimental and computational research has been conducted to study the subcutaneous absorption and bioavalibility (McDonald et al., 2010; Richter et al., 2012; Richter & Jacobsen, 2014; Bittner et al., 2018; Sánchez-Félix et al., 2020), a thorough understanding of the abosoption of mAbs delivered by subcutaneous injection is still lacking. In particular, the fluid dynamics, transport, absorption, and ensuing biomechanical processes that occur at the injection site during and after delivery are poorly understood (Sánchez-Félix et al., 2020).

Subcutaneous tissue is composed of adipocytes connected through a fibrous network that provides mechanical strength. The extracellular matrix is an interconnected porous network saturated with interstitial fluid that contains proteins and electrolytes. The transport of macromolecules in the subcutaneous space is primarily controlled by fluid flow, although molecular diffusion and the interaction of electrical charges also play a role. During subcutaneous injection, the fluid pressure in the interstitium increases, producing fluid flow from the injection point to the surroundings, which results in the dispersion of injected mAbs in interstitial tissue (Baxter & Jain, 1989). Although less known, the increasing interstitial fluid pressure also causes a drop of the fluid pressures in the nearby blood vessels and lymphatic capillaries, which is produced by a local expansion of the tissue. Driven by the fluid pressure differences, a portion of interstitial fluid enters the blood vessels and lymphatic capillaries carrying some of the injected mAbs and leading to the drug absorption (Levick & Michel, 2010). Because it is difficult for large molecules like mAbs to diffuse across the vessel walls under concentration differences (Swabb et al., 1974; Reddy et al., 2006), the absorption of mAbs is dominated by the fluid exchanges between interstitial tissue, blood vessels, and lymphatic vessels. It is often assumed that the flow rate between the interstitial tissue and lymphatic vessels is linearly dependent on the transmural pressure difference (Granger, 1984; Negrini & Moriondo, 2011). Similar linear relationships have also been used to describe the fluid exchanges between interstitial tissue and blood capillaries (Lee et al., 2019). From a biomechanics point of view, the material responses including the tissue deformation, the fluid pressure changes in interstitial tissue, blood vessels, and lymphatic vessels, as well as the fluid dynamics in the three compartments, determine the absorption of mAbs in subcutaneous injection. Physics-based biomechanical models represent a useful tool to study the biomechanisms in subcutaneous injection and provide critical information that is difficult to obtain from *in vivo* and *in vitro* experiments. During the past years, several poroelastic models have been applied to simulate large deformation of tissue during subcutaneous injection, such as linear poroelasticity (de Lucio et al., 2021), Ogden hyperelasticity (Leng, de Lucio et al., 2021), and poro-viscoelasticity (Leng, Ardekani et al., 2021). However, these models focus on the material response and fluid pressure change in interstitial tissue, and do not model the changes of fluid pressures in blood vessels and lymphatic vessels or the drug transport induced by the material responses. Drug transport coupled with tissue deformation is also considered in some studies (Han et al., 2021; Hou et al., 2021; Zheng et al., 2021a; Leng et al., 2022; Rahimi et al., 2022). Nevertheless, these studies apply a linear model to describe the lymphatic uptake assuming that the lymphatic fluid pressure is constant, and ignore changes in lymphatic pressure due to tissue deformation and fluid flow. A noteworthy model of nanoparticle delivery in several porous compartments in deformable solid tumors was proposed by Wirthl et al. (2020). However, a biomechanical model that couples tissue deformation, fluid dynamics in all compartments, drug transport in interstitial tissue, and drug absorption in the circulatory system and lymphatic system in the context of subcutaneous injection is still missing.

To describe the fluid dynamics in interstitial tissue, blood vessels, and lymphatic vessels in subcutaneous injection, we leverage the idea of multi-network poroelasticity (MPET). This model is obtained by extending Biot’s theory for single compartment poroelasticity. In the MPET model, the material is assumed to be a mixture of different compartments. Each compartment is treated as a porous medium with its own porosity, permeability, fluid pressure, and fluid velocity. MPET models have been used to study the fluid flow between arteries, capillaries, interstitial fluid networks, and veins in multiple biomechanical applications including hydrocephalus (Levine, 2008; Tully & Ventikos, 2011; Sobey et al., 2012), cerebral edema (Vardakis et al., 2016), Alzheimer’s disease (Guo et al., 2018; Vardakis et al., 2019) and subject-specific cerebral fluid transport (Guo et al., 2020). However, a model that couples MPET and solute transport equations in a deformable porous medium has not been fully demonstrated in current literature. Here, we propose such a model and call it multi-network poroelasticity and transport theory (MPET^2^).

In the context of subcutaneous injection, the MPET^2^ approach constitutes a multi-compartment model based on the physical principles of fluid dynamics and tissue biomechanics that provides a spatial resolution of the fluid flow, mechanics, and drug transport in the interstitium, blood vessels, and lymphatic vessels. In addition to offering insight into the mechanisms that govern absorption at the injection site, our methodology can be integrated into whole-body pharmacokinetic (PK) models, leading to a computational pipeline that feeds well-established PK models with high-fidelity information of the absorption at the injection site. The capability of the proposed model is illustrated simulating a high flow rate injection of mAbs in the subcutaneous tissue. The model describes the tissue deformation, pressure evolution in the interstitial tissue, blood vessel and lymphatic vessel compartments, as well as the absorption in blood vessels and lymphatic vessels, and the formation of the drug plume in different compartments. From our simulations, we obtained absorption kinetics results and compared them with experimental data of subcutaneous absorption of macromolecules obtained from the depot clearance tests.

## 2. Methods and materials: the MPET^2^ model

### 2.1. A Three-network poroelastic model for subcutaneous tissue

**Figure 1.**
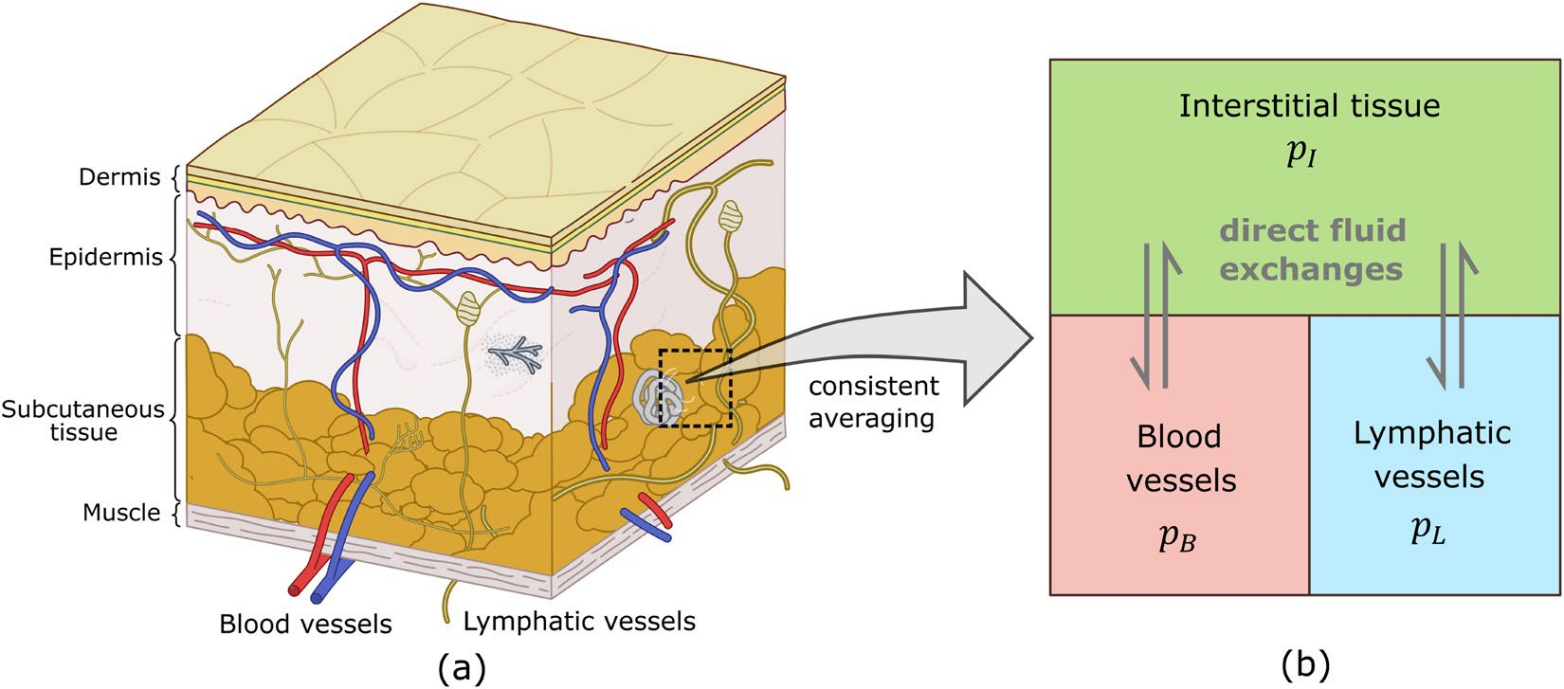
(a) Conceptual figure of the subcutaneous tissue with blood vessels and lymphatic vessels embedded in the tissue. (b) Schematic figure of the compartments considered in the MPET model for subcutaneous tissue.

Subcutaneous tissue is a highly heterogeneous biological system. As the innermost layer of skin, the tissue consists of mast cells, a fibrous network, fat, blood vessels, lymphatic vessels, and other components; see Figure 1(a). Instead of modeling each component explicitly, we model subcutaneous tissue as a mixture of three compartments, i.e. blood vessels, lymphatic vessels, and interstitial tissue. At a single point in the tissue, all compartments are simultaneously present with a particular volume fraction. Each compartment is treated as a continuous porous medium consisting of solid phase and fluid phase, but the fluid pressure, material properties, and porosity of each compartment are different; see Figure 1(b). The compartments are labeled with the subscripts B, L, and I that identify the variables related to blood vessels, lymphatic vessels, and interstitial tissue, respectively. There are fluid exchanges between communicating compartments that are driven by pressure differences. Under physiological equilibrium conditions, the model predicts constant fluid pressures satisfying $p_{B}>p_{I}>p_{L}$. This results in fluid flow from blood vessels to interstitial tissue and from interstitial tissue to lymphatic vessels. There is no direct fluid exchange between blood vessels and lymphatic vessels. Upon injection, the injectate changes the tissue stress and interstitial pressure, which in turn affect the convection of interstitial fluid and fluid flow from interstitial tissue to the other two compartments. The injected mAbs also follow the movement of interstitial fluid and enter the blood vessels and lymphatic vessels. The relationships between the tissue deformation, fluid dynamics in three compartments, and transport and absorption of mAbs, are detailed as follows.

### 2.2. Tissue poromechanics model

#### 2.2.1. Fluid flow and tissue deformation

The model describes the tissue deformation as well as the fluid dynamics in all compartments during and after injection. We assume that the entire drug dose is injected into the compartment that represents interstitial tissue. We treat the subcutaneous tissue as a homogeneous material. Under the assumption of small deformation, the Cauchy stress tensor is σ=λtr(ϵ)I+2μϵ, where $\epsilon =\frac{1}{2}(\nabla u+\nabla^{T}u)$ is the infinitestimal strain, u is the displacement field, *λ* and *μ* are Lame’s constants. The tissue displacement and pressures in the three compartments are obtained from the equations:

(1) $$\nabla \cdot \sigma =\alpha_{I}\nabla p_{I}+\alpha_{B}\nabla p_{B}+\alpha_{L}\nabla p_{L},$$

(2) $$\alpha_{I}\frac{\partial \epsilon_{v}}{\partial t}+w_{I}\frac{1}{M_{I}}\frac{\partial p_{I}}{\partial t}=w_{I}\nabla \cdot \left(\frac{\kappa_{I}}{\mu_{f}}\nabla p_{I}\right)+s_{B\to I}+s_{L\to I}+f_{I}+Q,$$

(3) $$\alpha_{B}\frac{\partial \epsilon_{v}}{\partial t}+w_{B}\frac{1}{M_{B}}\frac{\partial p_{B}}{\partial t}=w_{B}\nabla \cdot \left(\frac{\kappa_{B}}{\mu_{f}}\nabla p_{B}\right)+s_{I\to B}+f_{B},$$

(4) $$\alpha_{L}\frac{\partial \epsilon_{v}}{\partial t}+w_{L}\frac{1}{M_{L}}\frac{\partial p_{L}}{\partial t}=w_{L}\nabla \cdot \left(\frac{\kappa_{L}}{\mu_{f}}\nabla p_{L}\right)+s_{I\to L}+f_{L}.$$

Equation (1) is the linear momentum conservation of the mixture, and Equations (2)–(4) represent the mass balances of fluid in interstitial tissue, blood vessels, and lymphatic vessels, respectively. We assume that fluid flow follows Darcy’s law. We use the subscripts i,j=I,B,L to denote the different compartments. We call *p_i_* the fluid pressure; *α_i_* is the Biot–Willis coefficient satisfying $\phi_{s}\leq \alpha_{B}+\alpha_{L}+\alpha_{I}\leq 1$ where $\phi_{s}$ denotes the volume averaged porosity of the three compartments (Wang, 2017); $\epsilon_{v}=\text{tr}(\epsilon )$ is the volumetric strain; *w_i_* is the volume fraction of compartment *i* in the mixture; *M_i_* is the Biot–Willis modulus; *κ_i_* denotes the absolute permeability; *μ*_f_ denotes the fluid dynamic viscosity; $s_{i\to j}$ is the fluid exchange term between two communicating compartments; *f_i_* is a mass source that represents the physiological equilibrium conditions; and *Q* is a source term that models the injection.

#### 2.2.2. Fluid exchanges between communicating compartments

The fluid exchanges between communicating compartments are driven by the fluid pressure differences (Wiig & Swartz, 2012). Following Granger (1984) and Negrini & Moriondo (2011), we employ a linear relation between fluid flow and the pressure difference, such that

(5) $$s_{B\to I}=-s_{I\to B}=\xi_{\text{BI}}\left(p_{B}-p_{I}\right),$$

(6) $$s_{L\to I}=-s_{I\to L}=\xi_{\text{LI}}\left(p_{L}-p_{I}\right),$$

where *ξ*_BI_ and *ξ*_LI_ are the fluid interaction coefficients between compartments.

In subcutaneous tissue, fluid exchanges between compartments exist under physiological equilibrium conditions because the fluid pressures are different. To ensure that the pressures are constant under such conditions, mass source terms *f_i_* are introduced as:

(7) $$f_{I}=\xi_{\text{BI}}\left(p_{I}^{0}-p_{B}^{0}\right)+\xi_{\text{LI}}\left(p_{I}^{0}-p_{L}^{0}\right),$$

(8) $$f_{B}=\xi_{\text{BI}}\left(p_{B}^{0}-p_{I}^{0}\right),$$

(9) $$f_{L}=\xi_{\text{LI}}\left(p_{L}^{0}-p_{I}^{0}\right),$$

where the superscript 0 denotes the fluid pressures under physiological equilibrium conditions. Thus, for constant pressures in the three compartments, Equations (2)–(4) predict continuous fluid flow from the interstitium to lymphatic vessels and from blood vessels to the interstitium in accordance with those encountered in physiological conditions.

The constant pressures under physiological equilibrium conditions are used as initial pressures in the simulations. The average difference between the pressure of interstitial tissue and that of lymphatic vessels is ∼4–10 mmHg (Negrini & Moriondo, 2011). The pressure difference between blood vessels and interstitial tissue is ∼13 mmHg at the arterial end and ∼−7 mmHg at the venous end (Kurbel et al., 2001). Consistently with these data, in our simulations, we set the blood pressure to 1 kPa, interstitial pressure to 0 kPa, and lymphatic pressure to –1 kPa, respectively.

#### 2.2.3. Porosity change

We use the porosity to denote the volume fraction of fluid phase in each compartment. Porosity is affected by tissue deformation and fluid pressure. Following Dana et al. (2018) and Dana & Wheeler (2018) porosity is dependent on the volumetric deformation and fluid pressures, such that

(10) $$\phi_{i}=\phi_{i}^{0}+(\alpha -\phi_{s}^{0})(\epsilon_{v}-\epsilon_{v}^{0})+\frac{1-\alpha_{i}/w_{i}}{M_{i}}(p_{i}-p_{i}^{0})$$

where $\phi_{i}$ is the porosity of compartment *i*, namely, the volume of fluid in compartment *i* over the total volume of the compartment, $\phi_{i}^{0}$ is the initial porosity, $\phi_{s}^{0}=w_{I}\phi_{I}^{0}+w_{B}\phi_{B}^{0}+w_{L}\phi_{L}^{0}$ is the initial volume averaged porosity, and $\alpha =\alpha_{I}+\alpha_{B}+\alpha_{L}$ is the total Biot–Willis coefficient.

#### 2.2.4. Injection characterization

We model the injection as a source term in the mass balance of interstitial tissue. We follow the injection model proposed by de Lucio et al. (2021). The fluid coming from the needle is assumed to be distributed in a spherical region with radius $R_{\text{inj}}$, which is equal to the needle radius. The injection source term is dependent on time and space, such that

(11) $$Q(x,t)=Q_{0}Q^{D}(x)Q^{t}(t),$$

where $Q_{0}=V_{\text{inj}}/(T_{\text{inj}}\frac{4}{3}\pi R_{\text{inj}}^{3})$ is the constant injection flow rate with $V_{\text{inj}}$ denoting the total volume of injected drug and $T_{\text{inj}}$ denoting the duration of injection. The space-dependent function $Q^{D}(x)=\frac{1}{2}[1-\tanh(\Lambda (d(x)-R_{\text{inj}}))]$ ensures that the injected fluid is distributed in the spherical neighborhood of the injection point, where d(x) is the distance between x and the injection point. The time-dependent function $Q^{t}(t)=\frac{1}{2}\alpha_{T}[\tanh(\beta (t-t_{\text{sh}}))-\tanh(\beta (t-T_{\text{inj}}))]$ ensures that the injection occurs only in a certain time interval represented by $T_{\text{inj}}$. Table 1 lists values of parameters in the injection model, which correspond to an injection with a flow rate of 1440 mL/h (Shpilberg & Jackisch, 2013; Doughty et al., 2016).

**Table 1.** Values of parameters in the injection model.

Parameter	*α*_T_	*β*	*t*_sh_	Λ	$R_{\text{inj}}$	$V_{\text{inj}}$	$T_{\text{inj}}$
Value	10/9	7 s^–1^	0.5 s	5,000 mm^–1^	0.15 mm	2 mL	5 s

### 2.3. Drug transport and absorption model

The drug transport in each compartment and drug exchanges between communicating compartments are modeled by transport equations in deformable porous media (Zhang et al., 2012). In our MPET^2^ model the equations are:

(12) $$w_{I}\frac{\partial \left(\phi_{I}C_{I}\right)}{\partial t}+w_{I}\nabla \cdot \left(-\frac{\kappa_{I}}{\mu_{f}}\nabla p_{I}C_{I}\right)-w_{I}\nabla \cdot \left(\phi_{I}D_{I}\nabla C_{I}\right)=\Gamma_{\text{BI}}+\Gamma_{\text{LI}}+Q,$$

(13) $$w_{B}\frac{\partial \left(\phi_{B}C_{B}\right)}{\partial t}+w_{B}\nabla \cdot \left(-\frac{\kappa_{B}}{\mu_{f}}\nabla p_{B}C_{B}\right)-w_{B}\nabla \cdot \left(\phi_{B}D_{B}\nabla C_{B}\right)=-\Gamma_{\text{BI}},$$

(14) $$w_{L}\frac{\partial \left(\phi_{L}C_{L}\right)}{\partial t}+w_{L}\nabla \cdot \left(-\frac{\kappa_{L}}{\mu_{f}}\nabla p_{L}C_{L}\right)-w_{L}\nabla \cdot \left(\phi_{L}D_{L}\nabla C_{L}\right)=-\Gamma_{\text{LI}},$$

where the terms on the left side represent the rate of change of drug mass in the compartment, advection, and diffusion, respectively. The unknown $C_{i}=c_{i}/c_{\text{drug}}$ is the normalized concentration of mAbs where *c_i_* is the mass of mAbs per unit fluid volume in compartment *i* and $c_{\text{drug}}$ is the mass of mAbs per unit fluid volume in the injected solution, and *D_i_* is the diffusion coefficient, respectively. The parameter Γ*_ij_* represents the drug exchange between two communicating compartments. Because absorption of large molecules is dominated by fluid exchanges, we neglect the diffusion of mAbs due to concentration differences between communicating compartments. Following Dershowitz & Miller (1995), the drug absorption terms are defined as:

(15) $$\Gamma_{\text{BI}}=(1-d_{B\to I})s_{B\to I}C_{I}w_{I}\phi_{I}/\phi_{s}+d_{B\to I}s_{B\to I}C_{B}w_{B}\phi_{B}/\phi_{s},$$

(16) $$\Gamma_{\text{LI}}=(1-d_{L\to I})s_{L\to I}C_{I}w_{I}\phi_{I}/\phi_{s}+d_{L\to I}s_{L\to I}C_{L}w_{L}\phi_{L}/\phi_{s},$$

where $d_{B\to I}$ and $d_{L\to I}$ denote the directions of fluid exchanges, such that

(17) $$d_{B\to I}=\frac{1}{2}\left(1-\frac{s_{I\to B}}{\left|s_{I\to B}\right|}\right),$$

(18) $$d_{L\to I}=\frac{1}{2}\left(1-\frac{s_{I\to L}}{\left|s_{I\to L}\right|}\right).$$

Although our model does not include a size-exclusion mechanism, the parameters controlling the drug absorption into the circulatory and lymphatic systems can be selected based on the permeability of vessel walls and the molecule size.

### 2.4. Model parameters

The mechanical properties of subcutaneous tissue vary in experimental measurements. Recent publications show a wide range for the values of the poromechanical properties of subcutaneous tissue for different species and different sampling positions. Detailed discussions about the poromechanical parameters and transport parameters can be found in the study by de Lucio et al. (2021) and Rahimi et al. (2022). We use averaged values reported in literature for interstitial tissue of human subjects. However, there are few references covering the experimental measurements for mechanical properties of blood vessels and lymphatic capillaries embedded in subcutaneous tissue. In the absence of better information, we refer to validated MPET models in other human tissues and use similar values for the parameters of the two vessel compartments. Table 2 presents the values of the parameters used in this work.

**Table 2.** Values of the applied parameters.

	Parameter	Description	Value	Reference
Mechanical parameters	*E*	Young’s modulus	30 kPa	(Li et al., 2012; González-Suárez et al., 2015)
	*ν*	Poisson’s ratio	0.45	(Fung, 2013; Chen et al., 1996)
	*α*	Biot-Willis coefficient	0.99	(Podichetty et al., 2014; Podichetty & Madihally, 2014)
	*μ*_f_	Fluid viscosity	$10^{-3}$ Pa s	Water viscosity
Interstitial tissue	*M*_I_	Biot’s modulus	0.125 MPa	(Thomsen et al., 2014)
	*κ*_I_	Permeability	$6\times 10^{-13}$ m^2^	(Thomsen et al., 2014)
	$\phi_{I}^{0}$	Porosity	0.01	(Thomsen et al., 2014)
	*D*_I_	Drug diffusion coefficient	$10^{-11}$ m^2^/s	(Wright et al., 2018; Hung et al., 2019)
	$p_{I}^{0}$	Physiological fluid pressure	0 kPa	(Negrini & Moriondo, 2011; Kurbel et al., 2001)
	*w*_I_	Volume fraction	0.85	Estimated
	*α*_I_	Biot’s coefficient	0.8415	Estimated
Blood vessels	*M*_B_	Biot’s modulus	0.1 MPa	(Guo et al., 2018)
	*κ*_B_	Permeability	$1.2\times 10^{-12}$ m^2^	(Thomsen et al., 2014)
	$\phi_{B}^{0}$	Porosity	0.1	(Thomsen et al., 2014)
	*D*_B_	Drug diffusion coefficient	$10^{-11}$ m^2^/s	(Wright et al., 2018; Hung et al., 2019)
	$p_{B}^{0}$	Physiological fluid pressure	1 kPa	(Kurbel et al., 2001)
	*w*_B_	Volume fraction	0.1	Estimated
	*α*_B_	Biot-Willis coefficient	0.099	Estimated
Lymphatic vessels	*M*_L_	Biot’s modulus	0.1 MPa	(Guo et al., 2018)
	*κ*_L_	Permeability	$1.2\times 10^{-12}$ $m^{2}$	(Thomsen et al., 2014)
	$\phi_{L}^{0}$	Porosity	0.1	(Thomsen et al., 2014)
	*D*_L_	Drug diffusion coefficient	$10^{-11}$ m^2^/s	(Wright et al., 2018; Hung et al., 2019)
	$p_{L}^{0}$	Physiological fluid pressure	–1 kPa	(Negrini & Moriondo, 2011)
	*w*_L_	Volume fraction	0.05	Estimated
	*α*_L_	Biot’s coefficient	0.0495	Estimated
Fluid exchange coefficient	*ξ*_BI_	Exchange between interstitial tissue and blood vessels	$2.5\times 10^{-7}$ (Pa s)^–1^	(Michler et al., 2013)
	*ξ*_LI_	Exchange between interstitial tissue and lymphatic vessels	$1.25\times 10^{-7}$ (Pa s)^–1^	(Michler et al., 2013)

### 2.5. Numerical method

We solve the coupled system of Equations (1)–(4) and (12)–(14) using a monolithic algorithm. The spatial discretization is performed using Isogeometric Analysis, which provides higher accuracy than the classical finite element method (Hughes et al., 2005). We employ a second-order generalized-*α* approach for time discretization (Chung & Hulbert, 1993). In each time step, the nonlinear system of equations is solved utilizing a Newton–Raphson algorithm, and the resulting linear system is solved using the GMRES method (Saad & Schultz, 1986). The computational framework is implemented using the open source PETSc package (Balay et al., 1997; Abhyankar et al., 2018) and PetIGA (Dalcin et al., 2016). The algorithm was verified in a 2-D benchmark test with exact solution. We also performed mesh refinement studies to test the convergence of the algorithm. The two studies ensure that the proposed algorithm can provide us with stable and robust solutions of the governing equations.

## 3. Results and discussion

### 3.1. Model geometry, initial and boundary conditions

In this section, we perform a simulation of subcutaneous injection. The injected volume is 2 mL, and the injection time is 5 s, resulting in a flow rate of 1440 mL/h. We assume the injection process is axisymmetric and consider a cylindrical piece of subcutaneous tissue with a radius of 50 mm and a height of 50 mm. The simulation is performed in a cylindrical coordinate system. The bottom surface (*z* = 0 mm) is fixed, and the injection point is located on the symmetric axis (*r* = 0 mm) at 4 mm below the top surface. We also assume that the fluid can exit the tissue through the bottom surface and cylindrical surface (*r* = 50 mm). We use the boundary conditions:

(19) $$\left\{ \begin{array}{cc} & \nabla p_{i}\cdot n=0,\;u_{r}=0,\;\text{on}\;r=0\;\text{mm},\\ & p_{i}=p_{i}^{0},\;\;\mathrm{\sigma n}=0,\;\;\text{on}\;r=50\;\text{mm},\\ & p_{i}=p_{i}^{0},\;u_{z}=0,\;\;\text{on}\;z=0\;\text{mm},\\ & \nabla p_{i}\cdot n=0,\;\mathrm{\sigma n}=0,\;\text{on}\;z=50\;\text{mm}. \end{array}\right.$$

We employ a uniform mesh with 250,000 elements. We use a time step of Δt=0.001 s. The actual time simulated is 15 s.

We use the mass fraction of mAbs in blood vessels and lymphatic vessels to describe drug absorption. The drug concentrations *C_i_* used in Equations (12)–(14) represent the mass of drug in compartment *i* per unit volume of fluid in compartment *i*. To better compare the amount of drug in different compartments, we use the relative concentration

(20) $$C_{i}^{R}=C_{i}\phi_{i}w_{i}/\phi_{s},$$

which represents the mass of drug in compartment *i* per unit volume of fluid. The mass of mAbs in a compartment can be obtained as

(21) $$m_{i}^{\text{drug}}=\int_{\Omega }C_{i}^{R}\phi_{s}dv=\int_{\Omega }C_{i}w_{i}\phi_{i}dv,$$

where dv is the differential of volume of the mixture. The concentrations shown in the following results are the relative concentrations.

### 3.2. Material responses and fluid dynamics

**Figure 2.**
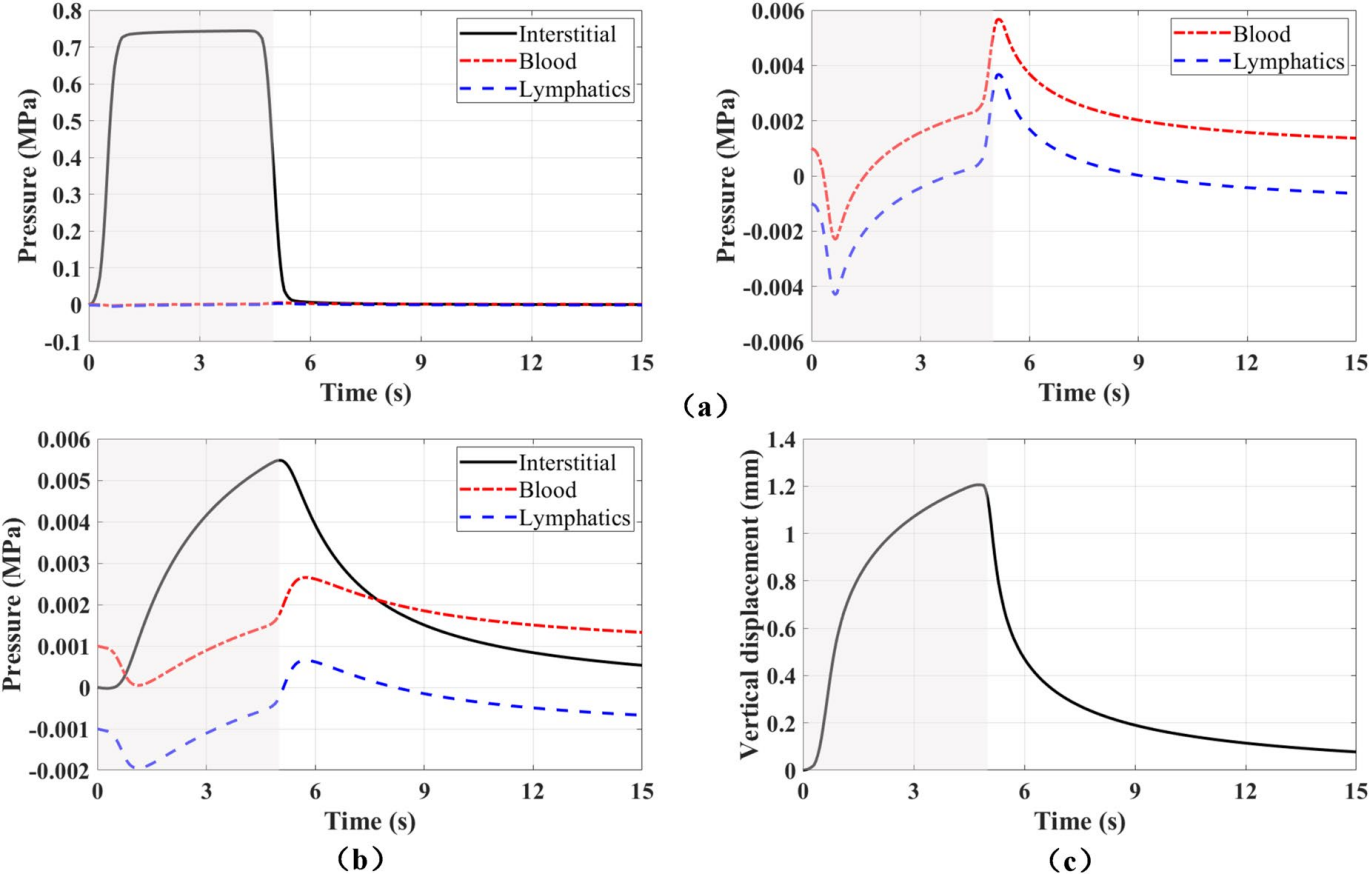
Time evolution of fluid pressures in different compartments at the injection point (a) as well as 10 mm below injection point (b), and vertical displacement on the top surface above the injection point (c) for an injected volume of 2 mL. The left figure in (a) shows the pressures in all compartments at the injection point, while the right figure shows the pressures in blood vessels and lymphatic vessels at the injection point (zoomed in). The shadow areas denote the injection time interval in all panels.

Figure 2 shows the time evolution of fluid pressures and vertical displacement. The pressure evolution at the injection point is depicted in Figure 2(a). The left panel shows the pressures in the three compartments, and the right panel shows details of the pressure evolution in the blood vessels and lymphatic vessels compartments. Because all the fluid is injected into interstitial tissue, the interstitial pressure increases significantly and reaches the maximum value of 0.74 MPa shortly after the injection begins. The injection also causes the local expansion of tissue, which results in the drop of pressures in blood vessels and lymphatic vessels at the beginning of the injection; see right panel. Afterward, the fluid flows into blood vessels and lymphatic vessels from interstitial tissue, and the fluid pressures in the two compartments increase. Similarly, near the end of injection the pressures in the blood and lymphatic vessel compartments show a sharp increase due to local contraction, and then decrease after the injection ends. The pressure differences between interstitial tissue and the two vessel compartments are highest at injection point, which causes the strongest fluid exchanges from interstitial tissue to the two compartments. However, because the flow rates between the compartments are much lower than the flow rate of the injection, the increments of the pressures in the two vessel compartments are much smaller. The maximum pressure increments in the two vessel compartments are approximately 5 kPa. Thus, the Darcy velocities of fluid that are driven by pressure gradients in the two vessel compartments are much smaller than that in interstitial tissue. Figure 2(b) illustrates the pressure evolution 10 mm below the injection point. Among the three compartments, the interstitial pressure has the largest increment since the Darcy velocity is highest in interstitial tissue. The pressures in blood vessels and lymphatic vessels first drop due to local expansion of the tissue, and then increase because of the fluid convection from the injection point and the local fluid exchanges. The curves of pressures in blood vessels and lymphatic vessels have very similar slope at all times. This observation suggests that the fluid convection from the injection point is the main driving force for the pressure increases in blood vessels and lymphatic vessels. The displacement evolution at the skin surface on top of the injection point is shown in Figure 2(c), with a value of 1.21 mm at the end of injection. The deformation decays quickly after injection. After 10 s from the injection end, the displacement is only 0.07 mm.

**Figure 3.**
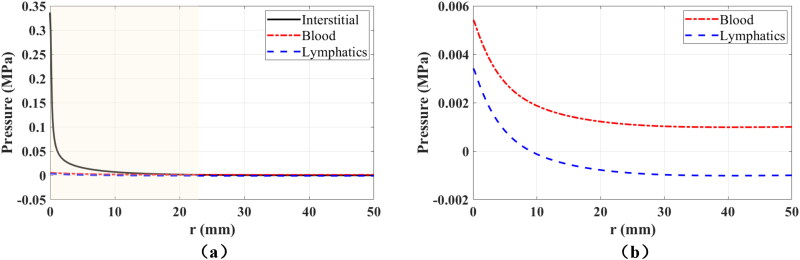
Radial distribution of fluid pressures in all compartments along the injection point at the end of injection (*t* = 5 s). All the fluid pressures are shown in (a), while only the pressures in blood vessels and lymphatic vessels are shown in the zoomed-in figure (b). The shaded region in (a) denotes the area where the pressure in interstitial tissue is highest among all compartments.

**Figure 4.**
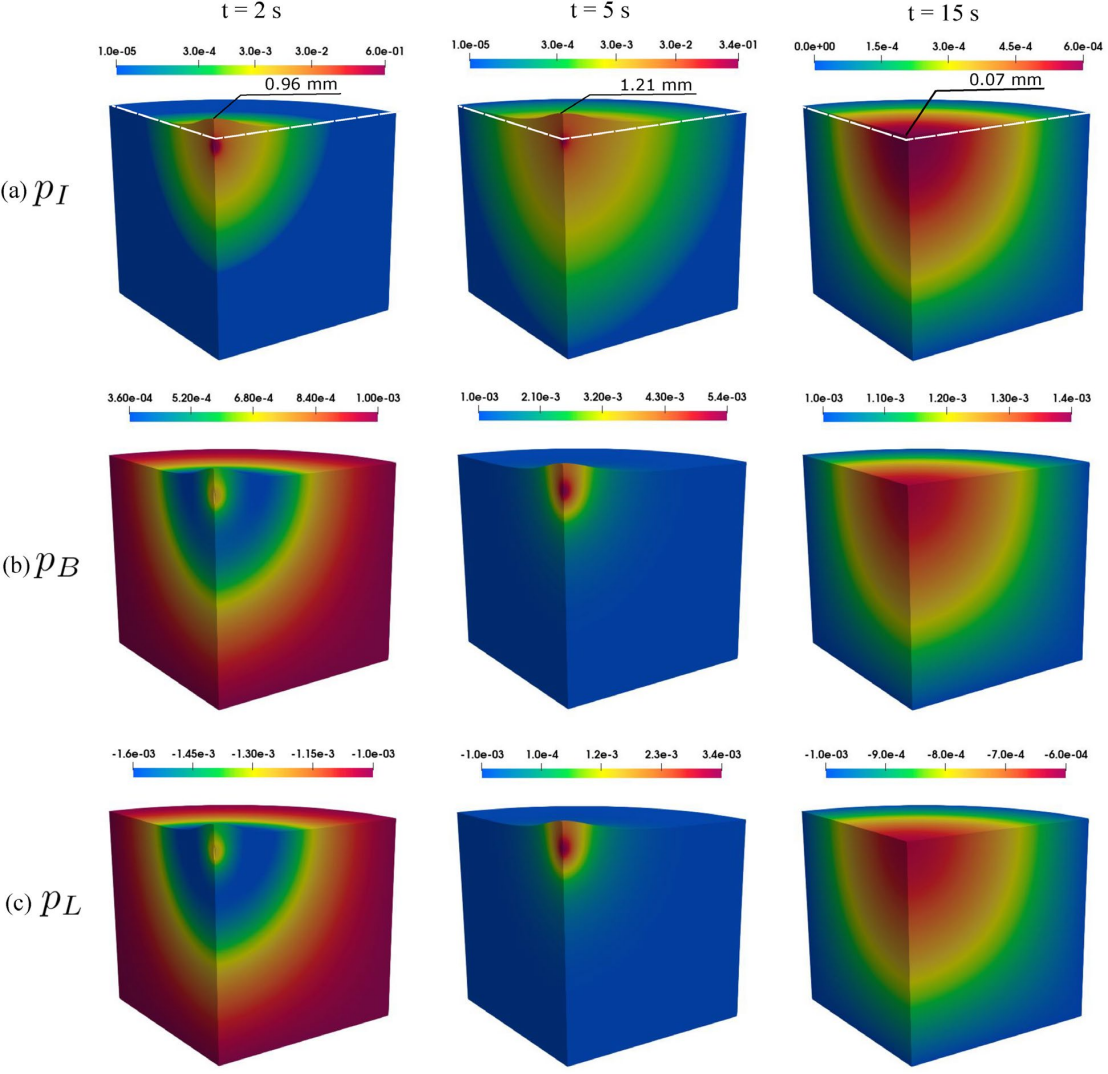
Spatial distribution of fluid pressures in interstitial tissue (a), blood vessels (b), and lymphatic vessels (c) at specific times for an injected volume of 2 mL. Unit for pressure: MPa. The largest vertical displacement is also depicted in (a). The white dash lines in (a) show the undeformed configuration. A scaling factor of 4 is applied to the displacement for easier visualization.

The spatial distributions of fluid pressures are critical for drug dynamics and absorption because they determine the fluid flow as well as the drug exchanges between compartments. Figure 3 shows the radial distribution of fluid pressures along the injection point at the end of injection (*t* = 5 s). The pressure in interstitial tissue is highly localized near the injection point, and the pressure gradient in interstitial tissue is much larger than in blood vessels and lymphatic vessels. At this time, the radius of the region where interstitial tissue has highest pressure is 22.9 mm. Inside this region, the communicating fluid flows from interstitial tissue to blood vessels and lymphatic vessels, carrying also the injected mAbs and resulting in drug absorption in the two compartments. However, the pressure differences between interstitial tissue and the other two compartments are much higher near the injection point due to the localized pressure distribution. For instance, the pressure differences are higher than 50 kPa at locations where *r* < 2 mm, which causes larger fluid exchanges and higher drug absorption. Figure 4 shows the spatial distribution of fluid pressures in all compartments as well as the displacement at specific times. The large expansion of tissue near the injection point leads to pressure drops in blood vessels and lymphatic vessels at the beginning of the injection; see *t* = 2 s in Figure 4. After that, fluid flows into the two vessel compartments from interstitial tissue, resulting in increasing fluid pressures and localized elevation of the pressure near the injection point at *t* = 5 s; see Figure 4. All pressures decay fast after the injection ends, as shown in the rightmost column of Figure 4.

**Figure 5.**
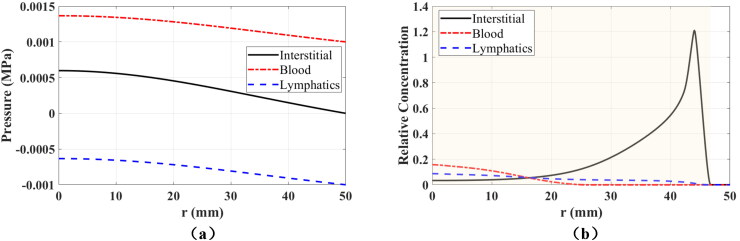
Radial distribution of fluid pressures (a) and relative concentrations (b) in all compartments along the injection point 10 s after the end of the injection. The shaded area in (b) shows the plume size in the interstitial tissue.

The radial distribution of fluid pressures along the injection point 10 s after the injection ends is depicted in Figure 5(a). At this moment, blood pressure is again the highest pressure across the entire tissue specimen, resulting in fluid exchanges from blood vessels to interstitial tissue. The fluid pressures in all compartments are close to the values under physiological equilibrium conditions. The maximum fluid pressures are still located at the injection point, but the maximum deviations from physiological values are only a little higher than 0.5 kPa in interstitial tissue and 0.4 kPa in the other two compartments. As a result, the strength of fluid exchanges is also similar to their values under physiological equilibrium conditions. In addition, the pressure gradients are very small, and there is almost no Darcy flow within the compartments.

### 3.3. Drug absorption and plume formation

**Figure 6.**
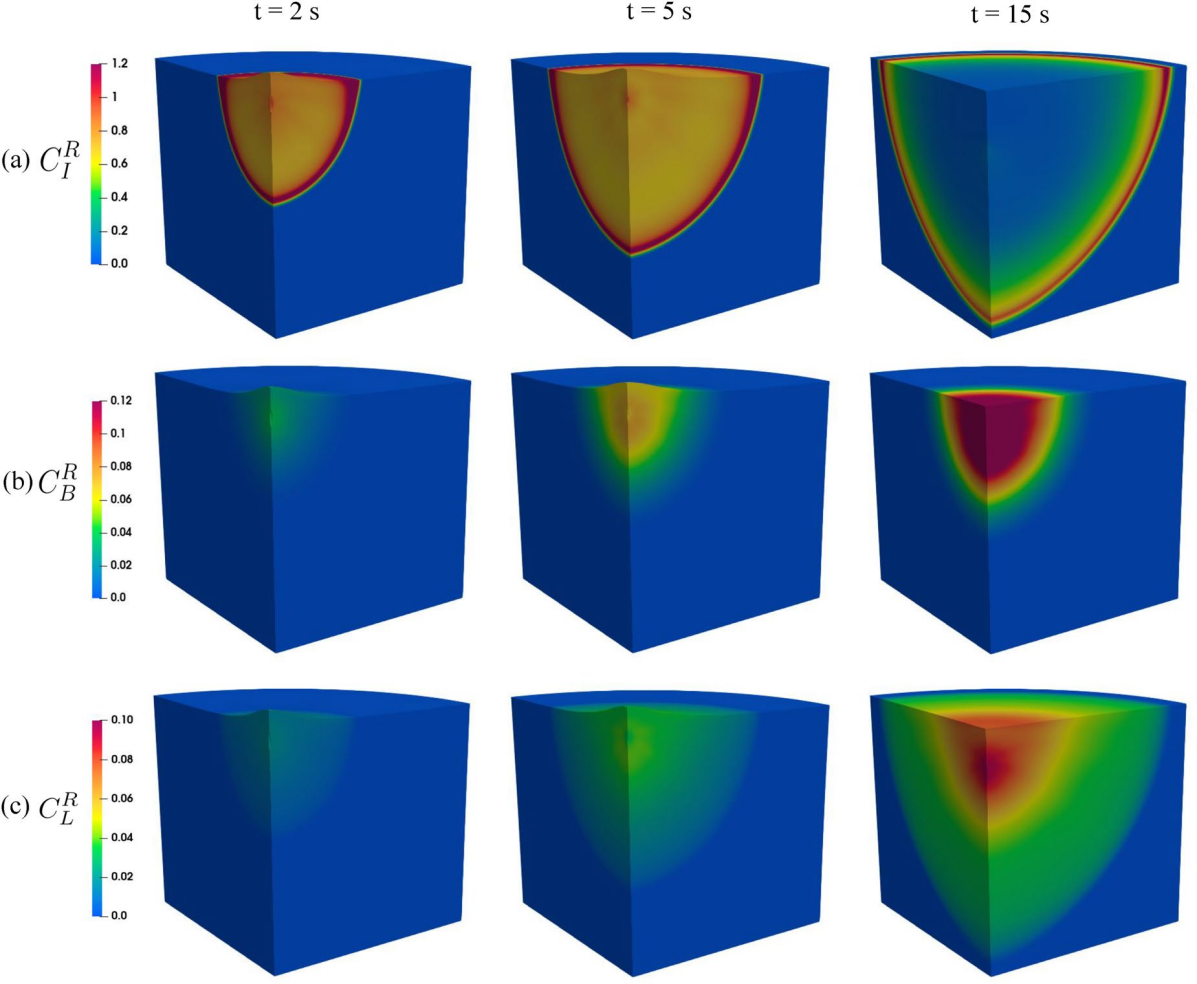
Spatial distribution of relative concentrations in interstitial tissue (a), blood vessels (b), and lymphatic vessels (c) at specific time steps under the injected volume of 2.0 mL.

Figure 6 illustrates the spatial distribution of relative concentrations in all compartments at different times. During the injection, the interstitial pressure is highly localized around the injection point, causing a massive fluid flow from the injection point to the surroundings. The injected drug is also transported to the surrounding tissue due to the fluid convection. Thus, the drug plume grows, and the drug concentration inside the plume remains fairly constant. Because the fluid is modeled as a weakly compressible liquid, the fluid is compressed at the edge of the plume where Darcy velocity is very small. The local compression of the fluid also leads to the accumulation of drug at the edge of the plume; see *t* = 2 s and *t* = 5 s in Figure 6(a). Meanwhile, most of the drug absorption occurs in the neighborhood of the injection point during injection because the pressure differences between compartments and the flow rates of communicating fluid are highest at such locations. There is no drug accumulation at the edge of the plumes in blood vessels and lymphatic vessels; see *t* = 2 s and *t* = 5 s in Figure 6(b,c). The region where interstitial pressure is higher than blood pressure is smaller than the plume in interstitial tissue, and only in this region the drug can be absorbed into blood vessels. In contrast, lymphatic uptake from interstitial tissue occurs in the entire interstitial plume. Thus, the size of the plume in the lymphatic compartment is close to that in interstitial tissue, and both are much larger than the plume size in blood vessels.

After injection, the pressures in all three compartments decay quickly and approach their physiological values. The blood pressure becomes the highest pressure across the entire tissue specimen, and the communicating fluid flows from blood vessels to interstitial tissue. A small portion of drug enters the interstitial tissue along with the fluid exchanges, and the drug fraction decreases a little in blood vessels. The flow from interstitial tissue to lymphatic vessels leads to drug absorption in the lymphatic system. Figures 5(b) and 6 illustrate, respectively, the radial and spatial distribution of relative concentrations 10 s after the injection ends. Both figures show large plumes that contain drug in interstitial tissue and lymphatic vessels. We also observe a steep drop in interstitial concentration at the edge of the plume because of the very small diffusion coefficient of mAbs. In blood vessels and lymphatic vessels where the Darcy’s velocities are smaller, the maximum concentration is located at the injection point, and there are no drug accumulations at the plumes edges. The plume size in the interstitial tissue, blood vessels, and lymphatic vessels are, respectively, 46.80 mm, 25.80 mm, and 46.35 mm.

### 3.4. Prediction of drug absorption and comparison with experiments

**Figure 7.**
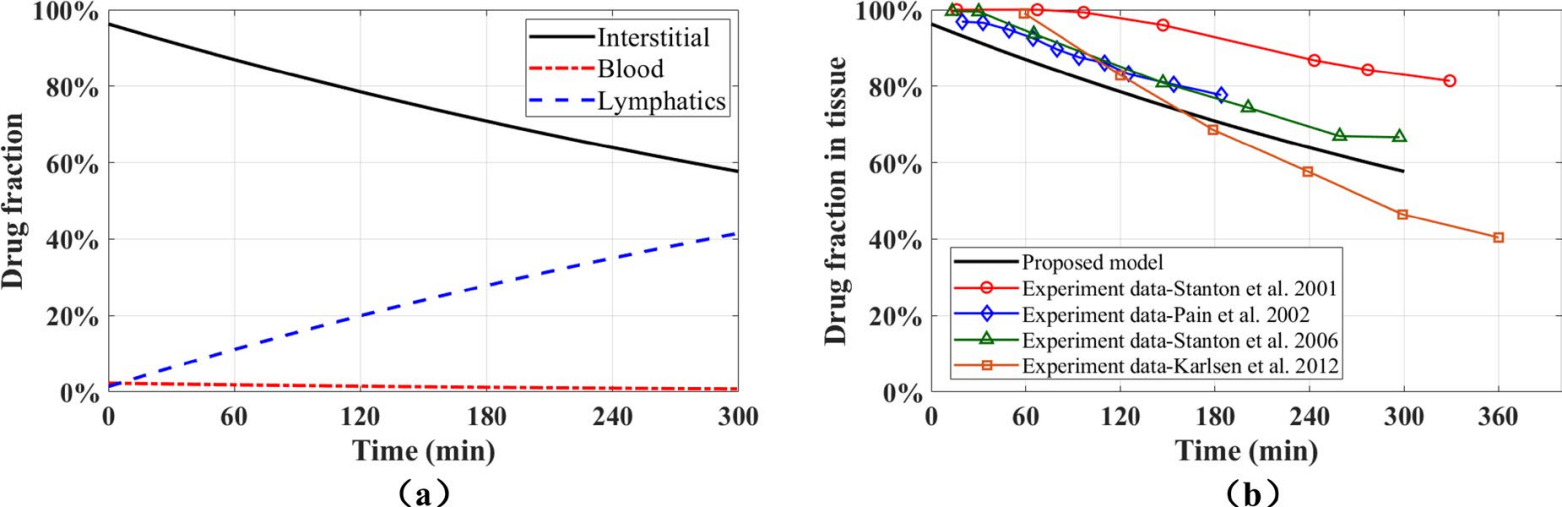
Prediction of the drug fractions for a time interval of 300 minutes. (a) Drug fractions in all compartments (model results). (b) Drug fractions in interstitial tissue – comparison between the MPET^2^ results and experimental data.

The physics-based simulation discussed so far describes in detail the fluid dynamics, transport, tissue deformation, and absorption processes at the injection site in the early stages. How the drug is distributed in the tissue and across the compartments during the injection plays a critical role in the overall absorption process (Sánchez-Félix et al., 2020). However, the majority of the absorption occurs after the injection ends. To simulate absorption in the time scale of several hours, we argue as follows: a few seconds after the injection ends, the fluid pressures and the rate of fluid exchange between compartments recover to their physiological values. The deformation of the tissue and Darcy velocities of fluid in all compartments are very small. Thus we can simplify the model by assuming that the subcutaneous tissue is in physiological equilibrium conditions. There is no displacement in the tissue, and the pressures are constant in all compartments. Darcy flow can be neglected, and the drug absorption is driven only by the fluid exchanges between communicating compartments. Under these assumptions, we only need to solve the transport equations, and use the concentration obtained from the full-scale, multiphysics simulation as the initial conditions for the transport equations. The evolution of drug fractions in all compartments in the 300 min following the injection is illustrated in Figure 7(a), and the drug fractions 300 min after injection are listed in Table 3. The drug fraction in blood vessels drops slowly due to the drug reabsorption into interstitial tissue, and the drug fraction in lymphatic vessels increases because of the constant fluid exchanges from interstitial tissue to lymphatic vessels. As more drug goes into the lymphatic system, the drug concentration in interstitial tissue decreases, and the drug absorption rate in the lymphatic system decreases as well.

**Table 3.** Predicted drug fractions in each compartment 300 minutes after injection.

	Interstitial tissue	Blood vessels	Lymphatic vessels
Drug fraction 300 min after injection	57.68%	0.80%	41.52%

To evaluate the proposed model, the predicted results are compared with several depot clearance tests in human subjects, which can be found in in literature (Stanton et al., 2001; Pain et al., 2002; Stanton et al., 2006; Karlsen et al., 2012). In these experiments, researchers labeled the injected protein with a radioactive tracer, and monitored the labeled drug for several hours after injection. The percentage of the drug remainning in the interstitial tissue is used as the parameter to measure the drug absorption. Figure 7(b) shows the comparison between the proposed model and the experimental data. Because the experiments are conducted at different locations of the body with different protein-based drugs, there are significant discrepancies between experimental data. However, all the experimental data suggest that the time evolution of the drug fraction remaining in the tissue after the injection (*M*_p_) can be described by the negative exponential relation $M_{p}=100e^{-kt}$, where *k* denotes the decay coefficient. The result of the proposed model also shows an exponential decay, and the calculated decay coefficient lies in the middle of all experimental data. While the model parameters could be adjusted to individually match the results for each of the molecules used in the experiments, some of the required molecule properties are not available for humans. There is also uncertainty on some of the model parameters including permeabilities and porosities of the compartments and further experiments are warranted. Note also that due to the absence of experimental data, the model neglects degradation at the injection site and binding to neonatal Fc receptor (FcRn).

## 4. Conclusion

We propose a multi-network poroelasticity and transport theory (MPET^2^) for subcutaneous injection of mAbs. The proposed biomechanical model provides predictions of the tissue deformation, the fluid velocity and pressure in interstitial tissue, blood vessels, and lymphatic vessels as well as the drug absorption in the three compartments after subcutaneous delivery of mAbs. The model spatially resolves all of these quantities providing high-fidelity information of the drug dynamics at the injection site that can be coupled with whole-body PK models to estimate bioavailability. We illustrate the capabilities of the proposed model by evaluating the drug absorption up to several hours after injection and comparing the results with experimental data obtained from depot clearance tests in human subjects. Due to the intensive computational cost, we include three compartments in the proposed model. However, the proposed model can be extended to more compartments. For instance, the blood vessels can be split into arterial vessels and venous vessels to better describe the physiological processes that occur in subcutaneous injection.

Although the model results show good agreement with experimental data, the model’s predictive capabilities may be limited by the uncertainty in some of the model parameters which are not readily available for human subjects. The Bayesian approach proposed by Tinsley Oden et al. (2017) coupled with inverse analysis could be used to address this limitation. Due to the absence of experimental data we neglected local degradation of the proteins, and FcRn binding, which may lead to overpredictions of the absorption. The MPET^2^ model, when coupled with whole-body PK models, may help understand the impact of local injection conditions on bioavailability, which is significant but remains poorly understood (Zheng et al., 2021b; Zou et al., 2021). This opens the opportunity to understand the effects on drug absorption and bioavailability of parameters such as injected fluid volume, injection flow rate, injection site, and forces applied to the tissue on the drug absorption. Other future efforts will include introducing nonlinear material models and multilayer models to better describe the mechanical response of the tissue.
